# Identification and validation of potential mRNA- microRNA- long-noncoding RNA (mRNA-miRNA-lncRNA) prognostic signature for cervical cancer

**DOI:** 10.1080/21655979.2021.1890377

**Published:** 2021-03-07

**Authors:** Jie Wang, Chen Zhang

**Affiliations:** Department of Obstetrics and Gynecology, The First Affiliated Hospital of Zhejiang Chinese Medical University, Hangzhou, China

**Keywords:** Cervical cancer, ceRNA network, prognostic signature

## Abstract

Cervical cancer is one of the most common causes of cancer deaths in women due to poor prognosis and high mortality rates. A novel mRNA-miRNA-lncRNA signature linked to prognosis of cervical cancer is needed to help clinicians judge the prognosis of individual patients more accurately. On the basis of GEO datasets, a total of 161 upregulated and 242 downregulated DE-mRNAs were identified firstly. Among them, eight potential biomarkers were found to have prognostic values with cervical cancer and miRNAs-lncRNAs related to these biomarkers were then analyzed to create mRNA-miRNA-lncRNA networks in cervical cancer. Moreover, in vitro experiments such as qRT-PCR, western blot and Edu assays were also performed to validate these promising targets. On the basis of these findings, a total of eight mRNA-miRNA-lncRNA subnetworks were finally established as a novel mRNA-miRNA-lncRNA signature and independent prognostic indicator of clinically relevant parameters by ROC analysis, univariate and multivariate Cox regression. Since some work of validation was done, it is believed that this mRNA-miRNA-lncRNA prognostic signature may be applied as a potential clinical judgment to estimate the prognosis of cervical cancer.

## GraphicalAbstract

**Figure:**
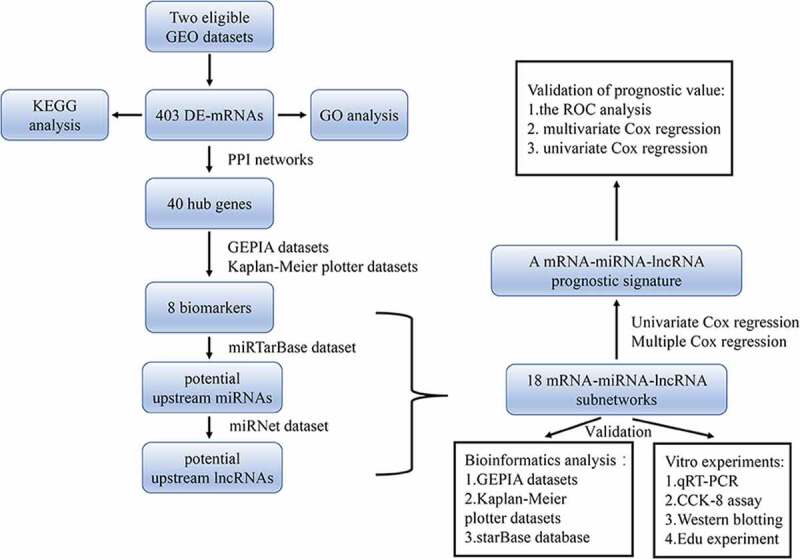

## 1. Introduction

Cervical cancer is the fourth common cause of cancer-related death in women and is the most commonly diagnosed cancer in 23 countries [1]. In transitioning countries, cervical cancer exhibits high incidence and mortality among younger people due to exposure to human papillomavirus (HPV), smoking, and immune-system dysfunction [2]. Although the huge improvement in treatment of cervical cancer has been achieved and effective prevention measures such as HPV screening and vaccination have been taken, the overall prognosis of women with invasive tumor remains poor [3]. Since the mechanism of cervical cancer progresses is not fully understood, our findings are expected to find out the molecular signature as well as fresh prognostic biomarkers for therapeutical targets of cervical cancer.

Noncoding RNAs (ncRNA) are RNAs that are not translated into proteins normally, including miRNAs, lncRNAs and circle RNAs [4], which regulate the expression of mRNA at both transcriptional and post-transcriptional levels [5]. There are a great many reports illustrating the implication of aberrantly expressed ncRNAs in tumorigenesis and promotion of cervical cancer [6–9]. In 2011, it was first proposed by Leonardo Salmena et al. that ncRNAs ‘communicate’ with each other during a ‘competing endogenous RNA (ceRNA)’ activity, and hence a network of regulation could be formed across the transcriptome [10]. Recently, growing studies about the mRNA-miRNA-lncRNA ceRNA networks indicated that ceRNA mechanism might play vital roles in the development of several cancers [11,12]. It is reported by Liu group that lncRNA XIST modulated the progression of thyroid cancer through the regulation of MET-PI3K-AKT signaling [13]. Non-small-cell lung carcinoma progression was promoted by H19 under the regulation of STAT3 signaling via sponging miR-17 [14]. Yang group found that LINC01133 sponged miR-106a-3p to modulate Wnt/β-catenin pathway and the expression of APC, which inhibited gastric cancer development [15]. However, identification of the key mRNA-miRNA-lncRNA networks greatly associated with cervical cancer prognosis is not sufficient.

In our study, several differentially expressed mRNAs (DE-mRNAs) of cervical cancer tissues with normal tissues in comparison were first selected by analyzing two Gene Expression Omnibus datasets (GSE29570 and GSE63514). According to the string database, protein–protein interaction (PPI) analysis was conducted to select top 40 hub genes. Taken the expression profiles and prognostic effects of hub genes into consideration, a total of seven upregulated genes and one downregulated hub gene were identified as biomarkers for following research. Then, miRTarBase [16] and miRNet database were utilized to predict the upstream regulatory miRNAs and lncRNAs. After that, the internetworks between these ncRNAs were subsequently found out based on the ceRNA theory, and their functional roles in the progression of cervical cancer were validated both in silico and in vitro. Finally, a fresh new mRNA-miRNA-lncRNA signature was constructed by univariate Cox regression, multivariate Cox regression to unveil promising biomarkers or targets therapies valuable for prognosis of cervical cancer and provide specific information to assist clinicians in judging patients for adjuvant therapy more appropriately.

## 2. Materials&methods

### 2.1 Datasets selection

It is well known that the Gene Expression Omnibus (GEO) (http://www.ncbi.nlm.nih.gov/geo/) is a database that contains chips, next-generation sequencing, and other high-throughput sequencing data. Mesh terms ‘cervical cancer’ and ‘human’ were used to search the GEO dataset and all the results were further filter with ‘Expression profiling by array’ and ‘Homo sapiens’. In consequence, 125 datasets were achieved. To guarantee the reliability of our study, datasets that met the following terms were excluded:

1. Less than 30 samples

2. Using only cell lines, organoids or peripheral blood of patients

As a result, the datasets from GSE29570 (including 17 healthy samples and 45 cervical cancer samples) and GSE63514 (including 24 healthy samples and 28 cervical cancer samples) were selected for subsequent analyses.

### 2.2 Differential genes expression analysis

The detailed contents of datasets were downloaded from the GEO datasets. The ‘limma’ Bioconductor R package was used to test differential expression [17]. Quantile normalization was used to normalize the data in gene expression microarrays, which assured the statistical distribution of each sample is the same [18]. After setting the cutoff criteria as adjust P < 0.05 and |log2FC| >1, DE-mRNAs were selected from the two datasets. Then, Venn diagrams were plotted by VENNY 2.1.0 (http://bioinfogp.cnb. csic.es/tools/venny/index.html). The commonly presented intersection DE-mRNAs in GSE29570 and GSE63514 datasets were re-declared as key DE-mRNAs, including upregulated and downregulated key DE-mRNAs.

### 2.3 GO/KEGG pathway analysis

In order to forecast the possible functions of those key DE-mRNAs, Kyoto Encyclopedia of Genes and Genomes (KEGG) pathway and Gene ontology (GO) analyses were conducted through the Database for Annotation, Visualization and Integrated Discovery (DAVID). Besides, ggplot2 package of R software [19] was further used to visualize the top enriched KEGG pathways and GO terms with P < 0.05.

### 2.4 Identification of hub genes

Search Tool for the Retrieval of Interacting Genes (STRING) database [20] was utilized to construct the specific PPI networks between the DE-mRNAs. After inputting all the DE-mRNAs into the database, the PPI pairs were visualized in the network if a combined confidence score ≥0.4. Then, as a handy plug-in of Cytoscape software, CytoHubba [21] was used to verify the significant hub genes in the constructed PPI networks by measuring the degree of DE-mRNAs connectivity. Based on the node degree in the Cytoscape software, the top 20 hub genes of the commonly DE-mRNAs were selected.

### 2.5 Gene expression validation

As a newly developed interactive server, The Gene Expression Profiling Interactive Analysis (GEPIA) is known for its functions to analyze 9736 neoplasms and 8587 healthy samples from The Genotype-Tissue Expression (GTEx) project and The Cancer Genome Atlas (TCGA) [22]. In our study, expressions of hub genes and lncRNAs in cervical cancer were analyzed (P < 0.05) by GEPIA, which contains 306 samples of cervical cancer and 13 samples of normal tissues.

### 2.6 Survival data analysis

In this study, all the prognostic roles of hub genes and lncRNAs were evaluated by using Kaplan-Meier plotter [23]. Sources for the Kaplan-Meier plotter databases include GEO, The European Genome-phenome Archive (EGA) and TCGA. Kaplan-Meier plot of survival was made to compare results between patient cohorts and the hazard ratio with 95% CI and log rank P-value were calculated. The expression of hub genes and lnRNAs of cervical cancer were screened in this database. Log-rank P-value < 0.05 was considered to have statistical significance.

### 2.7 Prediction of key miRNAs and lncRNAs

MiRTarbase is a database collection of target interactions with miRNAs which are confirmed experimentally by weak evidence (microarray, western blot and reporter assay) and strong evidence (next-generation sequencing experiment and qPCR) [16]. Key miRNAs of hub genes were screened through miRTarbase in our study. To obtain more convinced predictions, merely miRNA-target interactions validated by strong evidence (microarray, western blot and reporter assay) were included. For our study, the interaction between lncRNAs and key miRNAs was predicted through the miRNet database, a tool friendly in use for studies associated with miRNA [24,25]. The setting of ‘target type-lncRNAs’ and ‘Organism-H.sapies’ was displayed as selection criteria. Furthermore, the expression levels of these predicted key lncRNAs were assessed by GEPIA database as mentioned above.

### 2.8 Co-expression analysis

StarBase database is a public platform along with gene expression data of various types of cancers, which is derived from 10,882 RNA-seq and 10,546 miRNA-seq data in TCGA project, allowing researchers to perform RNA-RNA correlation analysis [26,27]. The interrelations of mRNA-miRNA, mRNA-lncRNA and miRNA-lncRNA pairs in cervical cancer were assessed by starBase v3.0 and P < 0.05 was considered to have statistical significance.

### 2.9 Cell culture

The Hela cell line was purchased from the cell bank of the Chinese Scientific Academy and was cultured in DMEM medium (Gibco, Life Technologies, USA) with 10% fetal bovine serum (Biological Industries, USA) with 1% Penicillin-Streptomycin at 37◦C in a humidified incubator containing 5% CO2.

### 2.10 Small interfering RNA & qRT-PCR

Particular siRNAs purchased from GenePharma (China) were used to transfect Hela cell line with lipofectamine2000 (Invitrogen, USA). Two days later, the cells were harvested for further experiments.

TRIzol reagent (Invitrogen, USA) was used to extract RNA from the cell lines. Then, RNA was reversely transcribed into cDNA by using a Reverse Transcription Kit (Takara, China). SYBR Green (Takara, China) was used for real-time PCR analysis through biosystems 7500/7500 fast real-time PCR system. Put the reaction solution into the PCR amplification instrument and perform PCR amplification according to the procedure. All the detailed components of reaction solution and procedure are listed in supplementary table 6–7. The results were normalized to the expression of glyceraldehyde-3-phosphate dehydrogenase (GAPDH). The comparative CT (2− ΔΔCT) method was used to determine the relative levels of CCDC144NL-AS1, hsa-miR-18a-5p, hsa-miR-221-3p, hsa-miR-19a-3p and ERS1 versus GAPDH.

Two siRNAs each were designed to target CCDC144NL-AS1:

siRNA-1, 5ʹ-GGAAUUGGUGAUUGGCUUTT-3ʹ;

siRNA-2, 5ʹ-CCUGUACAUCCUUACCUAUTT-3ʹ.

And qRT-PCR analysis was conducted by the SYBR-Green method. Primer sequences were as listed below:

GAPDH:5ʹ-TGCACCACCAACTGCTTAGC-3ʹ(forward),

5ʹ-GGCATGGACTGTGGTCATGAG-3ʹ(reverse);

CCDC144NL-AS1:5ʹ-ACATTTGGCTACACAGGGAAGA-3ʹ(forward),

5ʹ-CATTGCTCAGGTCCTTCACTCA-3ʹ(reverse);

hsa-miR-18a-5p:5ʹ-TGTCGCCTTCTCTCTGACCC-3ʹ(forward),

5ʹ-GCCAGGCTAACCAAGAAAAAGG-3ʹ(reverse);

hsa-miR-221-3p:5ʹ-GTGTGTGAGTGGCGGTCT-3ʹ(forward),

5ʹ-GCCAGCCTCAGCTTAATCCA-3ʹ(reverse);

hsa-miR-19a-3p:5ʹ-TGTGAAGCCTGTAGCTTGGAA-3ʹ(forward),

5ʹ-AGATGGCCCCATTGGACATT-3ʹ(reverse);

ERS1:5ʹ-AGGACAACACAAAGATCTGCAA-3ʹ(forward),

5ʹ- CCCTGTTGCTAAGCCGATGA-3ʹ(reverse);

### 2.11. Cell vitality

The cervical cancer cells with or without transfection were plated in 96-well plates (3*10^4^ cells per well with 200ul culture medium) and cultured at 37◦C in a humidified incubator containing 5% CO2. Cell vitality was evaluated by Cell Counting Kit-8 (Beyotime, China) at 1–5 days following the manufacture instructions. The absorbance value was measured at 450 nm after 1 h of incubation (37◦C in incubator containing 5% CO2).

### 2.12 Western blotting

Total protein from cervical cancer cells was extracted using cell lysis buffer. The BCA method was used to detect the protein concentration. Western blotting serves as a foundational experiment technique which undergoes three main procedures: 1. Compounding gel electrophoresis to separate proteins by 4–20% Express PLUSTMPAGE gels (GenScript, USA); 2. Transferring proteins to a polyvinylidene difluoride (PVDF) membrane; 3. Selecting immunodetection of a specific antigen. All the antibodies were obtained from Abcam (Cambridge, UK): anti-GAPDH, anti-ERS1. Finally, electrochemiluminescence (ECL) was used to read and the immunoblots were examined with a visible imaging system.

### 2.13 Edu experiment

Edu experiment was utilized to detect the cell multiplication ability. After adding DAPT and DMSO or AdNICD and AdControl for 72 h, the waste medium was discarded and the experiment was conducted according to the EdU kit instructions (RiboBio, China, each group has 3 holes). Samples were observed under a fluorescent microscope.

$$Risk\;core\;=\;\overset{n}{\underset{i=1}{\sum }}coefiXid$$

### 2.14 Construction of prognostic signature in cervical cancer

Based on the clinical parameters of cervical cancer from the TCGA datasets, univariate Cox proportional hazard regression (PHR) analysis was utilized to identify all the ncRNAs linked with prognosis (P < 0.001). Then a prognostic signature was established through multivariate Cox PHR analysis. The risk score of every patient was calculated on the basis of RNAs expression profiles using the following formula:

All cervical cancer patients were divided into high-risk group and low-risk group based on the median risk score. Multivariate Cox regression and univariate Cox regression analysis were utilized to assess the prognosis with grade, age, T stage and risk score.

### 2.15 Statistical analysis

A large part of the statistical analysis has been technically done by the aforementioned bioinformatic resources. The R software (version3.4.1) was used for all statistical analyses. For the screening of DE-mRNAs in GEO datasets, Hochberg False Discovery Rate and Benjamini method were taken in to modify the P values. A two-tailed t-test was used to estimate differential expressions of mRNA, miRNA and lncRNA. Fisher’s test was widely used to identify the significant GO terms as well as KEGG pathways. It was considered that P < 0.05 had statistical significance.

## 3. Results

In order to explore promising targets valuable for prognosis of cervical cancer, various public datasets were used to find 18 mRNA-miRNA-lncRNA subnetworks. Based on the ceRNA hypothesis, the correlations of all these mRNAs, miRNAs and lncRNAs were then determined with significant value of expression and prognosis in cervical cancer, which were also validated by vitro experiments. Moreover, an mRNA-miRNA-lncRNA prognostic signature was further established and confirmed as important prognostic indicator in cervical cancer by using the ROC analysis, multivariate and univariate Cox regression analysis. It is believed that this mRNA-miRNA-lncRNA prognostic may help clinicians estimate the prognosis of patients in cervical cancer more accurately.

### 3.1 Identification of DE-mRNAs in cervical cancer

**Figure 1.**
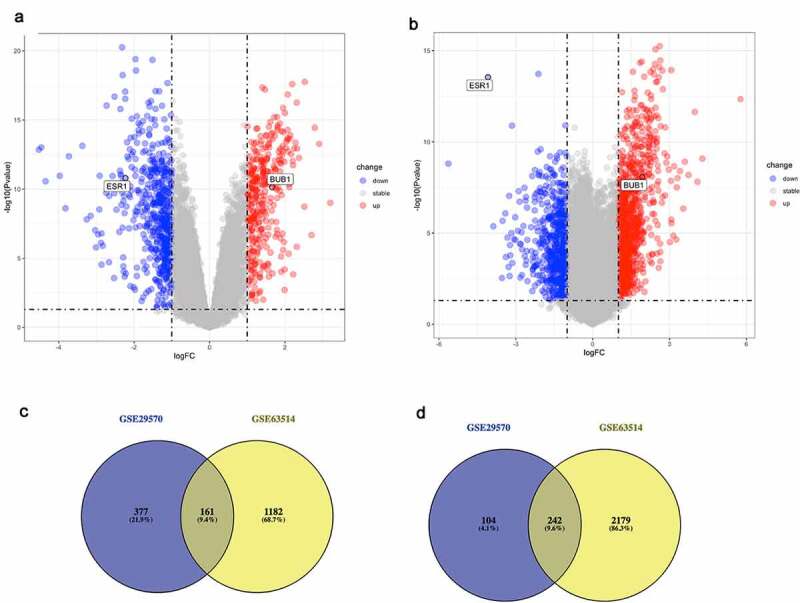
Identification of DE-mRNAs in two GEO datasets

Two independent GSE dataset (GSE29570 and GSE63514) of cervical cancer that fulfilled criteria were selected for the further analysis. Threshold value was set as P < 0.05 and |log2FC| >1 to identify DE-mRNAs in these two datasets (Figure 1(a-b)). For the GSE29570 dataset, 538 upregulated genes and 346 downregulated genes were selected. And in the GSE63514 dataset, 1343 upregulated and 2421 downregulated genes were selected out. Finally, 403 DE-mRNAs, including 161 upregulated and 242 downregulated significant DE-mRNAs, were identified by intersecting upregulated genes or downregulated genes separately (Figure 1(c-d)). Details of all DE-mRNAs were listed in Supplementary Table 1.

**Table 1.** The correlations between miRNA-mRNA/lncRNA-mRNA pairs identified by starBase database

miRNA	mRNA/lncRNA	R	P-value
hsa-miR-18a-5p	FTX	−0.193	6.72E-04
hsa-miR-18a-5p	MALAT1	−0.112	4.95E-02
hsa-miR-18a-5p	CCDC144NL-AS1	−0.161	4.76E-03
hsa-miR-18a-5p	LINC01089	−0.137	1.68E-02
hsa-miR-18a-5p	PSMA3-AS1	−0.151	8.27E-03
hsa-miR-18a-5p	RBPMS-AS1	−0.235	3.27E-05
hsa-miR-18a-5p	MEG3	−0.14	1.46E-02
hsa-miR-18a-5p	LINC01278	−0.188	9.27E-04
hsa-miR-18a-5p	SH3BP5-AS1	−0.174	2.21E-03
hsa-miR-18a-5p	MIR4697HG	−0.135	1.82E-02
hsa-miR-18a-5p	CRNDE	−0.234	3.44E-05
hsa-miR-18a-5p	CKMT2-AS1	−0.21	2.11E-04
*hsa-miR-18a-5p	LINC00667	−0.189	9.12E-04
hsa-miR-19a-3p	CCDC144NL-AS1	−0.199	4.71E-04
hsa-miR-19a-3p	RBPMS-AS1	−0.258	−2.58E-01
*hsa-miR-19a-3p	MSC-AS1	−0.15	8.48E-03
hsa-miR-19a-3p	LINC00467	−0.143	1.24e-2
hsa-miR-19a-3p	CRNDE	−0.164	3.96E-03
hsa-miR-19a-3p	ZEB1-AS1	−0.169	2.95E-03
hsa-miR-221-3p	CCDC144NL-AS1	−0.199	−1.99E-01
hsa-miR-221-3p	RBPMS-AS1	−0.258	5.00E-06
hsa-miR-19a-3p	CCDC144NL-AS1	−0.199	4.71E-04
FTX	ESR1	0.226	1.69E-02
MALAT1	ESR1	0.192	8.55E-03
CCDC144NL-AS1	ESR1	0.129	1.69E-02
LINC01089	ESR1	0.137	8.55E-03
PSMA3-AS1	ESR1	0.150	1.69E-02
RBPMS-AS1	ESR1	0.16	8.55E-03
MEG3	ESR1	0.259	1.69E-02
LINC01278	ESR1	0.203	8.55E-03
SH3BP5-AS1	ESR1	0.334	1.69E-02
CCDC144NL-AS1	ESR1	0.129	2.43E-02
RBPMS-AS1	ESR1	0.16	5.12E-03
LINC00467	ESR1	0.215	1.46E-04
CRNDE	ESR1	0.133	2.02E-02
ZEB1-AS1	ESR1	0.133	2.02E-02
CRNDE	ESR1	0.133	2.02E-02
ZEB1-AS1	ESR1	0.191	1.91E-01
CCDC144NL-AS1	ESR1	0.129	2.43E-02

*Pairs that don’t meet the competing endogenous RNA hypothesis

(a-b) The volcano plots of DE-mRNAs in GSE29570 and GSE63514 datasets. The horizontal axis indicates −10(adj P. Val), and the vertical axis indicates log FC. All of the DE-mRNAs were shown on the two volcano plots: the gray dots represent genes with no differentially expression, and the blue dots and red dots respectively represent the downregulated and upregulated genes. (c-d) The intersection of upregulated and downregulated DE-mRNAs of two datasets

### 3.2 GO analysis of the intersected differentially expressed mRNAs

**Figure 2.**
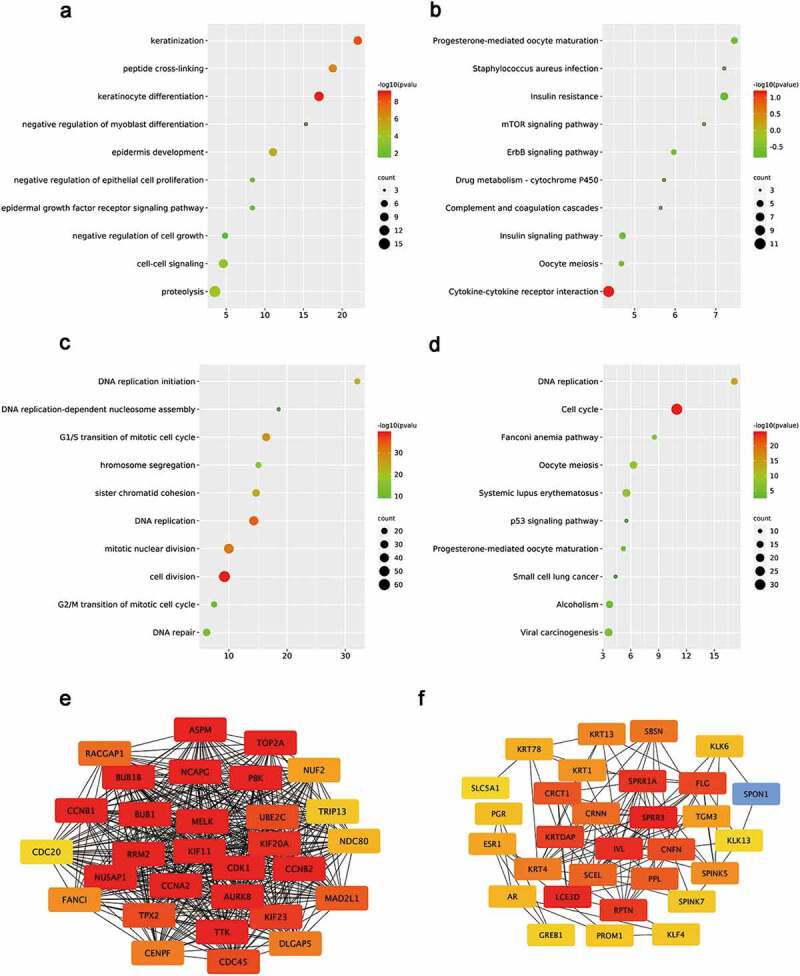
Functional analysis for the DE-mRNAs and identification of hub genes in protein-protein network

The intersected DE-mRNAs was further analyzed by the Gene Ontology (GO) annotation and KEGG pathway analyses. GO analyses were conducted from three distinguished aspects: biological process (BP), cellular component (CC) and molecular function (MF) for downregulated and upregulated DE-mRNAs. According to the results, upregulated DE-mRNAs were significantly enriched (P < 0.05) in terms of cell-cell signaling, proteolysis, negative regulation of cell growth, keratinocyte differentiation (Figure 2(a)). As shown in Figure 2(b), the analysis of KEGG pathway exhibited that upregulated DE-mRNAs were enriched (P < 0.05) in some cancer-related pathways such as ErbB signaling pathway, mTOR signaling pathway [28] and insulin signaling pathway. For downregulated DE-mRNAs, the results showed that downregulated DE-mRNAs were significantly enriched in activities associated with cell division and proliferation, such as DNA replication initiation, sister chromatid cohesion, G2/M transition of mitotic cell cycle, G1/S transition of mitotic cell cycle and chromosome segregation (Figure 2(c)). What’s more, KEGG pathway analysis of downregulated DE-mRNAs also exhibited multiple malignancy-associated pathways including p53 signaling pathway, Fanconi anemia pathway, small cell carcinoma and viral carcinogenesis (Figure 2(d)). Moreover, the results of CC and MF items enrichment were selected and ranked by gene counts (Supplementary Figure 1).

(a-b) Enriched BP process of the upregulated and downregulated significant DE-mRNAs. (c-d) Enriched KEGG pathways of the upregulated and downregulated significant DE-mRNAs. (e-f) The top 20 hub genes of the significantly upregulated genes and downregulated genes respectively

### 3.3 Establishment and analysis of PPI network

To further investigate the correlative interactions of the identified DE-mRNAs, PPI networks were established respectively for the downregulated DE-mRNAs and upregulated DE-mRNAs. Our results in Supplementary Figure 2 show that the complicated and close interactions among mRNAs are more obvious in upregulated DE-mRNAs. According to the node degree calculated by the cytoscape software, the top 20 DE-mRNAs in two dysregulated gene groups were identified as hub genes and visualized in Figure 2(e-f). In subsequence, those 40 hub genes were selected for further analysis (Supplementary Table 2).

### 3.4 Validation of expression pattern and survival analysis for hub genes

**Figure 3.**
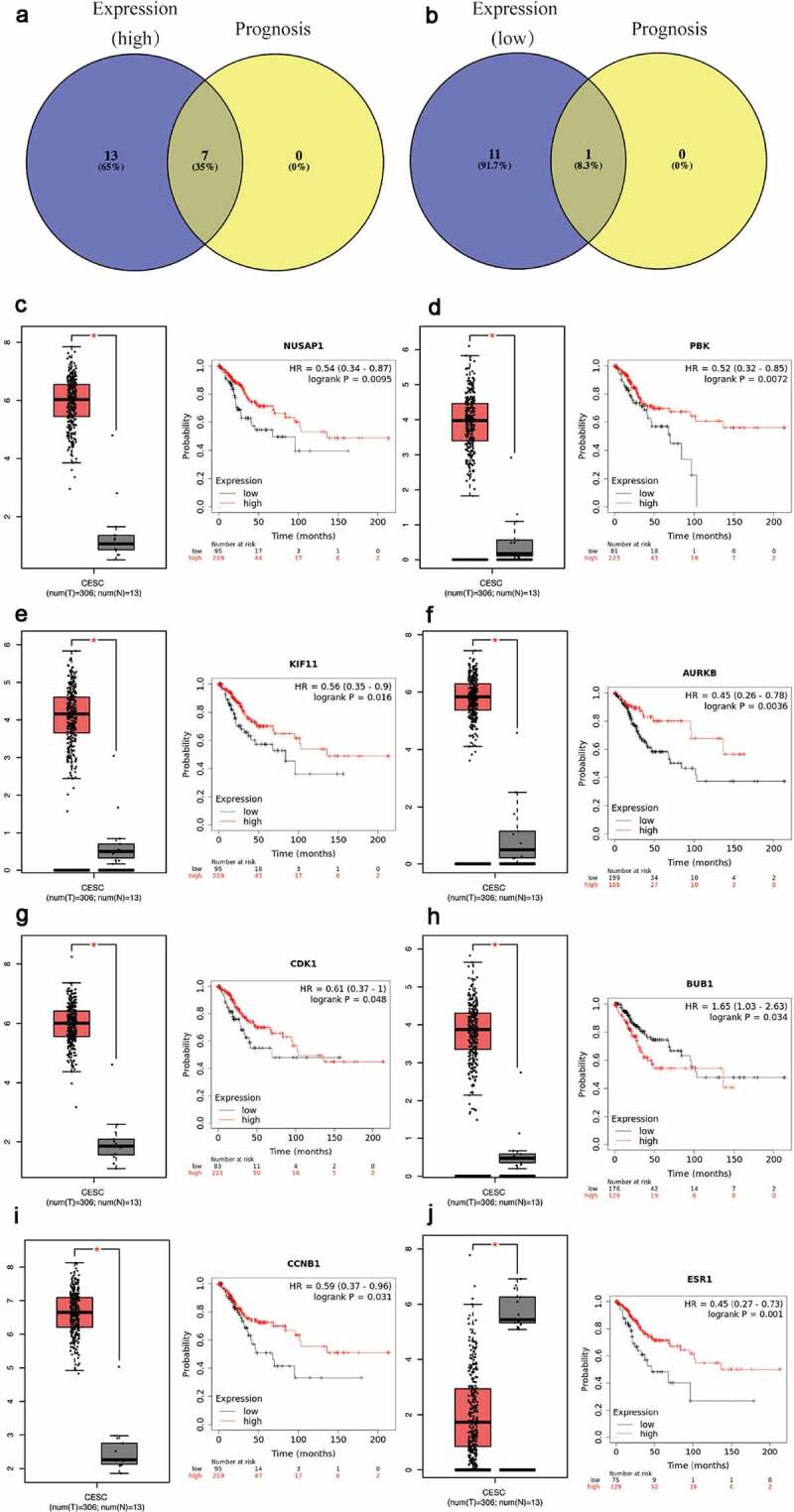
Screening the biomarkers in cervical cancer

**Figure 4.**
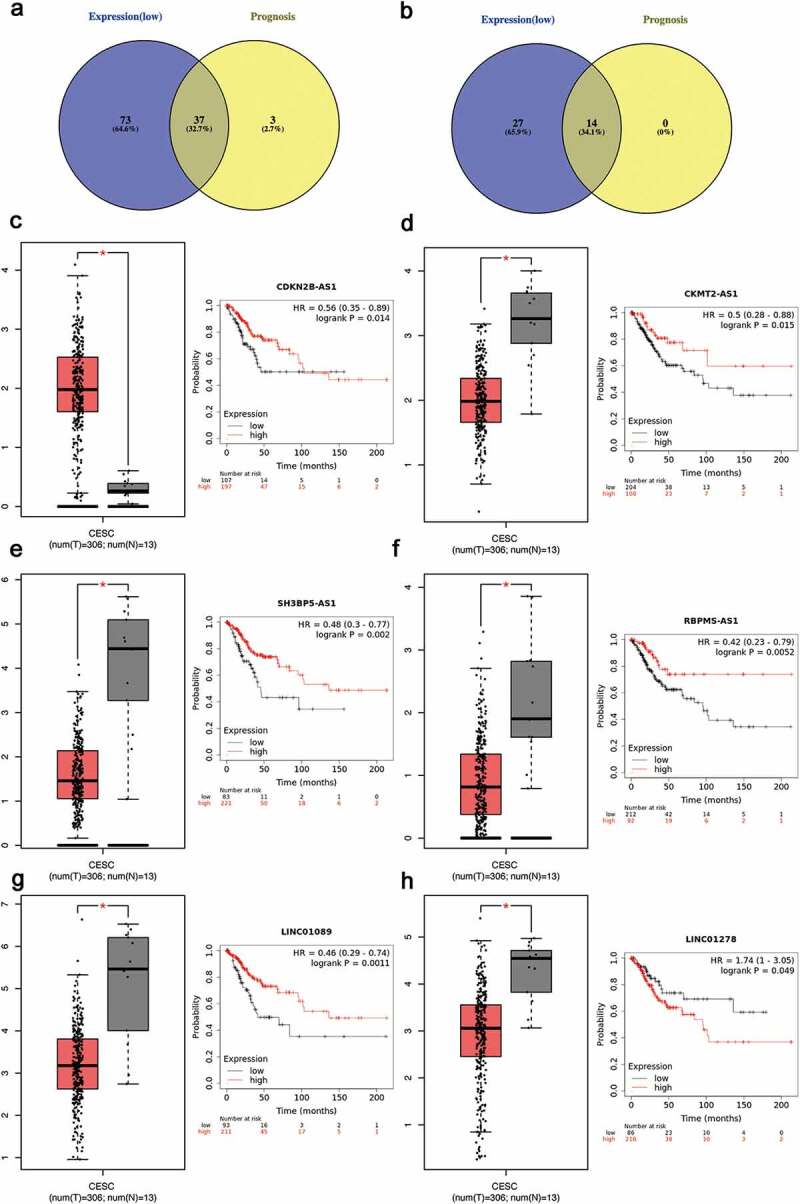
Screening the key lncRNA in cervical cancer

The expression of top 40 hub genes was further performed in GEPIA database to validate the expression of hub genes in cervical cancer. Meanwhile, Kaplan–Meier plotter database was used to elucidate the prognostic values of those hub genes for overall survival in cervical cancer patients. After combination of the expression pattern and survival analysis, eight upregulated hub gens (CCNB1, BUB1, CDK1, AURKB, KIF11, PBK and NUSAP1) stood out with dramatical upregulation in cervical cancer and were significantly correlated with good prognosis of cervical cancer (P < 0.05, Figure 3(a)). On the other hand, there was only one downregulated hub gene (ESR1) with low expression and poor prognosis in cervical cancer patients (P < 0.05, Figure 3(b)). All the survival curves and expression boxplots were exhibited in Figure 3(c-j) and Supplementary Table 3. Furthermore, the seven upregulated hub genes and one downregulated hub gene which meet criteria of expression pattern and survival prognosis were identified as biomarkers for next analyses.Figure 4(b-c)

(a-b) Identification of biomarkers among the predicted mRNAs by combining expression and prognosis analyses using GEPIA and Kaplan Meier-plotter databases, respectively. (c-j) The representative expression and prognostic value of biomarkers validated in GEPIA and Kaplan Meier-plotter databases

(a-b) Identification of key lncRNAs among the predicted lncRNAs by combining expression and prognosis analyses using GEPIA and Kaplan Meier-plotter databases, respectively. (c-h) The representative expression and prognostic value of potential lncRNAs validated in GEPIA and Kaplan Meier-plotter databases

### 3.5 Identification and validation of upstream miRNAs and lncRNAs

As a miRNA-target interactions database, miRTarBase was utilized to predict potential upstream miRNAs of the biomarkers and only miRNA-target interactions proved by strong evidence (western blot, qPCR or reporter assay) were included. In Supplementary Table 4, a total of 41 potential miRNAs were predicted to regulate seven biomarkers (CCNB1, BUB1, CDK1, AURKB, PBK, NUSAP1 and ESR1). Potential upstream miRNA of KIF11 was absent. The theory that lncRNA interacts with miRNA as ceRNA by competing for regulating mRNA [29,30] is widely accepted. MiRNet was used to predict the potential upstream lncRNAs. As a result, a total of 113 potential upstream lncRNAs in the database were predicted for 41 potential miRNAs (Supplementary Figure 3). According to the ceRNA theory, the eligible lncRNA should be positively correlated with mRNA. Thus, the expressions pattern and prognostic values of these lncRNAs in cervical cancer were further validated with the help of GEPIA database and Kaplan-Meier plotter database. As a consequence, a total of 37 upregulated lncRNAs and 14 downregulated lnRNAs had significant value of expression and prognosis in cervical cancer samples with normal controls as a comparison. Some survival curves and expression boxplots are exhibited in Figure 3(c-h).

### 3.6 Construction and validation of lncRNA-miRNA-mRNA interactional networks in cervical cancer

**Figure 5.**
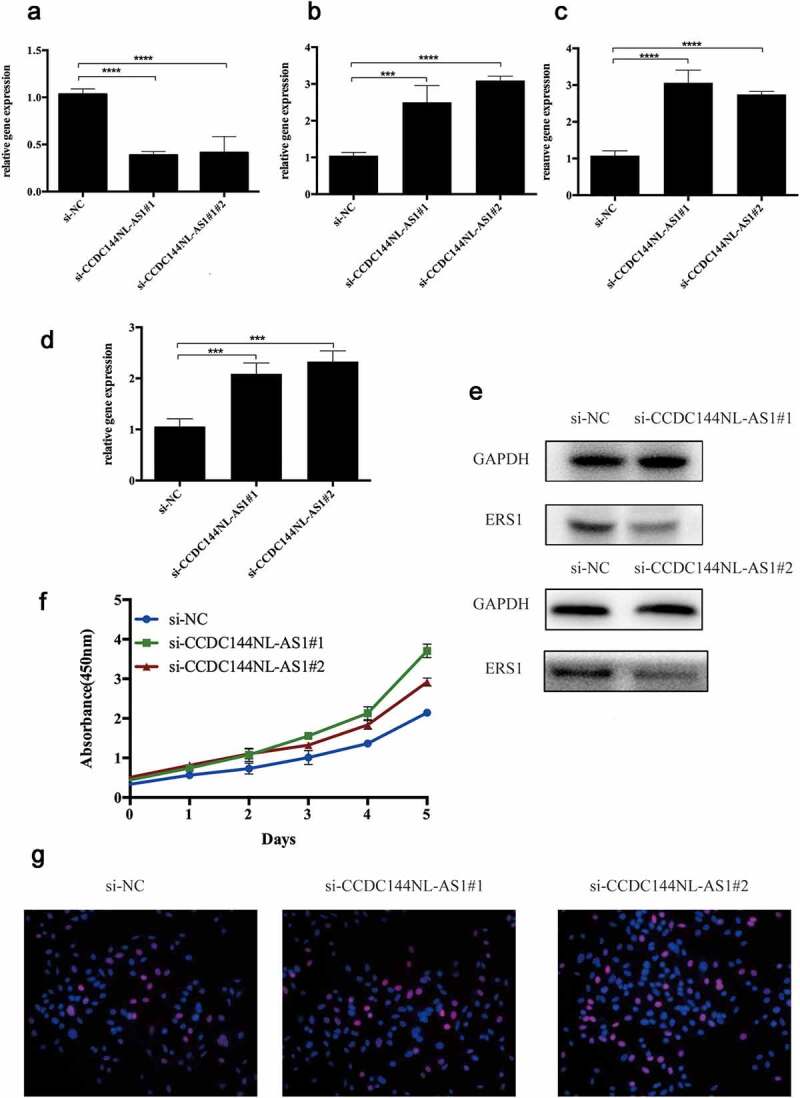
Validation of ce-RNA network

**Figure 6.**
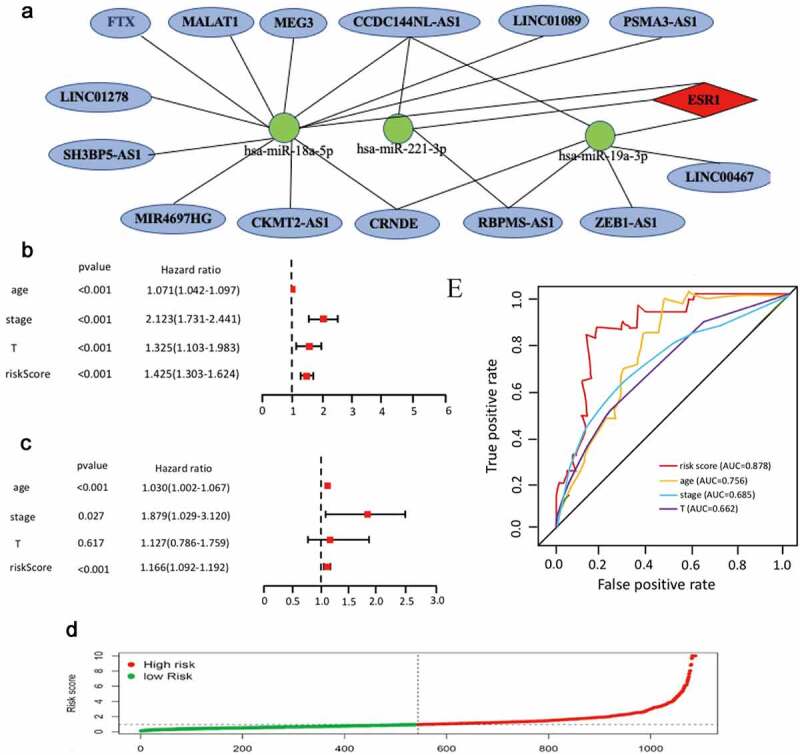
The Cox regression analysis for evaluating the independent prognostic value of the risk score

According to the ceRNA hypothesis mentioned above, miRNAs have an opposite co-expression relationship with mRNAs and lncRNAs, whereas lncRNAs have a positive co-expression relationship with mRNA. Thus, we assessed associtaions between all these RNA interaction pairs by using the starBase database and all of the RNA co-expression results consistent with ceRNA hypothesis were exhibited in Table 1. As shown in Figure 5, the network totally contained three mRNA-miRNA pairs (ESR1-miR-18a-5p, ESR1-miR-19a-3p,ESR1-miR-221-3p), 18 miRNA-lncRNA pairs (FTX-hsa-miR-18a-5p, MALAT1-hsa-miR-18a-5p, MEG3-hsa-miR-18a-5p, CCDC144NL-AS1-hsa-miR-18a-5p, LINC01089-hsa-miR-18a-5p, PSMA3-AS1-hsa-miR-18a-5p, LINC01278-hsa-miR-18a-5p, SH3BP5-AS1-hsa-miR-18a-5p, MIR4697HG-has-miR-18a-5p, CKMT2-AS1-hsa-miR-18a-5p, CRNDE-hsa-miR-18a-5p, LINC00467-hsa-miR-19a-3p, ZEB1-AS1-hsa-miR-19a-3p, RBPMS-AS1-hsa-miR-19a-3p, CRNDE-hsa-miR-19a-3p, CCDC144NL-AS1-hsa-miR-19a-3p, RBPMS-AS1-hsa-miR-221-3p, CCDC144NL-AS1-hsa-miR-221-3p) and 14 lncRNA-mRNA pairs (FTX-ESR1, MALAT1-ESR1, CCDC144NL-AS1-ESR1, LINC01089-ESR1, PSMA3-AS1-ESR1, RBPMS-AS1-ESR1, MEG3-ESR1, LINC01278-ESR1, SH3BP5-AS1-ESR1, LINC00467-ESR1, CRNDE-ESR1, ZEB1-AS1-ESR1, MIR4697HG-ESR1, CKMT2-AS1-ERS1). Taken all these results into consideration, 18 pairs of ceRNA subnetwork were finally identified (Figure 6(a)), which fully met the rule of ceRNA hypothesis.

(a) Hela cell was transfected with two CCDC144NL-AS1 siRNAs. qRT-PCR was used to detect the transfection efficiency. (b-d) qRT-PCR was used to detect the expression of hsa-miR-18a-5p, hsa-miR-221-3p and hsa-miR-19a-3p (e) The expression of ERS1 was analyzed by Western blotting (f) CCK-8 assays were conducted to examine Hela cell viability after the knockdown of CCDC144NL-AS1 (g) Edu assays were used to examine cell proliferation (red signal). The cell nuclei were counterstained with Hoechst (blue signal). Representative images are shown

(a) The potential mRNA-miRNA-lncRNA regulatory network related to cervical cancer prognosis. The red diamond in the network represented ERS1. The green round in the network represented miRNA. The blue one in the network represented lncRNA. (b-c) The univariate and multivariate Cox regression analysis of risk score, age, grade and T stage. (c) Calculate the AUC for risk score, age, grade, and T stage of the total survival risk score according to the ROC curve

To further improve the credibility of our findings, we chose three subnetworks (CCDC144NL-AS1-hsa-miR-18a-5p-ESR1, CCDC144NL-AS1-hsa-miR-221-3p- ESR1, CCDC144NL-AS1-hsa-miR-19a-3p-ESR1) to validate our hypothesis. After the downregulation of CCDC144NL-AS1 (Figure 5(a)), the expression of hsa-miR-18a-5p, hsa-miR-221-3p and hsa-miR-19a-3p were significantly increased (Figure 5(b-d)). Moreover, CCK8 was further conducted to show that si-CCDC144NL-AS1 cervical cancer cells exhibited higher proliferative capacity. Then, Western blotting and Edu experiments were also performed to demonstrate that the downregulation of CCDC144NL-AS1 in cervical cancer cells inhibited the expression of ESR1 and increased cell proliferative ability. Taken all the above into consideration, these results indicated the oncogenic roles of these ceRNA networks in the progression of cervical cancer.

### 3.7 Construction of mRNA-miRNA-lncRNA prognostic signature

On the basis of 18 mRNA-miRNA-lncRNA subnetworks of cervical cancer (Figure 6(a)), univariate Cox regression was used to analyze expression profiles of the18 RNAs. A total of 11 RNAs were identified with the fulfillment of criterion (P < 0.001) (Supplementary Table 5). Furthermore, multiple Cox regression analysis was used to identify eight RNAs (CCDC144NL-AS1, RBPMS-AS1, CRNDE, hsa-miR-18a-5p, hsa-miR-19a-3p, hsa-miR-221-3p and ESR1) to construct a mRNA-miRNA-lncRNA prognostic signature (Table 2). According to the expression of these eight RNAs in each cancer samples, the risk score was calculated as follows. Risk score = 0.36*hsa-miR-18a-5p + 0.28*hsa-miR-19a-3p + 0.3* hsa-miR-221-3p – 0.14* CCDC144NL-AS1 – 0.04* RBPMS-AS1 – 0.12* CRNDE – 0.11* LINC01089 – 0.21* ESR1. In addition,

**Table 2.** The expression levels of these eight RNAs

Name	Coef	HR	P-Value
CCDC144NL-AS1	−0.13621	0.834773	4.92E-02
RBPMS-AS1	−0.03891	0.783722	2.12E-02
CRNDE	−0.12139	0.792313	4.83E-02
LINC01089	−0.11290	0.76392	4.93E-02
hsa-miR-18a-5p	0.357782	1.233472	5.07E-04
hsa-miR-19a-3p	0.276341	1.123782	6.08E-04
hsa-miR-221-3p	0.302148	1.271231	1.34E-02
ESR1	−0.21023	0.882372	6.73E-04

all cancer samples were divided into low-risk group and high-risk group based on the median risk score. The risk curve was performed to show that the risk coefficient of patients in the low-risk group was lower than patients in the high-risk group (Figure 6(d)). Multivariate and univariate Cox regressions were then utilized to confirm our mRNA-miRNA-lncRNA signature as an important prognostic indicator for cervical cancer. The 95% CI and the hazard ratio (HR) of risk score in univariate Cox regression analysis were1.303–1.624 and 1.425 (P < 0.001). The 95% CI and the hazard ratio (HR) of risk score in multivariate Cox regression analysis were 1.092–1.192 and 1.166 (P < 0.001). All the results suggested that the mRNA-miRNA-lncRNA signature was an important prognostic indicator in cervical cancer (Figure 4(b-c)). Receiver operating characteristics (ROC) analysis was also performed to confirm the specificity and sensitivity of risk score on the prognostic of cervical cancer. The area under the ROC curve (AUC) of the risk score was 0.878 (Figure 4(e)), suggesting our mRNA-miRNA-lncRNA prognostic signature in patients with cervical cancer had good specificity and sensitivity.

## 4. Discussion

With 342,000 deaths and 604,000 new cases worldwide in 2020, cervical cancer is the leading cause of cancer death in 36 countries with high incidence and poor prognosis in females [1,31]. Despite great advances in the surgery, radiotherapy and precautions of cervical cancer have been achieved [32], it is important to solve the problems with cervical cancer of high risks and poor prognosis in patients by exploring fresh new prognostic indicators.

Increasing studies have demonstrated that abnormally expressed ncRNAs, such as miRNAs and lncRNAs, may be major contributors involved in pathogenesis and progression of cervical cancer [33–36]. Based on the ceRNA theory [10], accumulating evidence also suggested that ceRNA networks participate in tumorigenesis, tumor development and other pathological processes of cancer, providing a new idea for the clinical prediction of cervical cancer prognosis. For instance, Rui X et al. elucidated that targeting miR-637/RING1 axis could help lncRNAC5orf66-AS1 promote cell proliferation in cervical cancer [37]. Circle RNA has-circ-0000515 was reported to upregulated the expression of ELK1 functioning as a miR-326 sponge to promote cervical cancer development [38]. Nevertheless, comprehensive and systematic and analysis of ceRNAs in cervical cancer is imperative.

In our study, a specific novel ceRNA signature with prognosis was successfully established in cervical cancer by way of lncRNA-miRNA-mRNA pattern. To the best of our knowledge, this is the first study to identify the specific ceRNA network in cervical cancer by way of ‘mRNA-miRNA-lncRNA’ order pattern. Inspiringly, each RNA in the ceRNA network indicated a significant prognostic value in cervical cancer, which may provide some alternative biomarkers and potential therapeutic targets. Firstly, a total of 161 upregulated and 242 downregulated DE-mRNAs were identified by intersection of two GEO datasets (GSE29570 and GSE63541). The GO analysis unveiled that these DE-mRNAs were greatly enriched in some cancer-related GO items such as cell adhesion [39], cell-matrix adhesion [40], regulation of cell cycle and angiogenesis [41]. In addition, KEGG pathway enrichment analysis also proved that these significant DE-mRNAs had association with mTOR signaling pathway and p53 signaling pathway, which regulated the invasion and metastasis of cervical cancer [42,43]. These results suggested that DE-mRNAs identified through intersection of GEO datasets may play important roles in cervical cancer.

In order to explore more integrated relationships and specific functions of these significant DE-mRNAs in cervical cancer, PPI networks were constructed by using the STRING database, which showed complicated associations among these DE-mRNAs especially in upregulated group. Based on widely accepted knowledge that genes with more node degree in the PPI network usually play more roles, the top 40 hub genes were identified in the two PPI networks according to node degree. Then, the top 40 hub genes were conducted to evaluate the expression and prognostic values of cervical cancer and 7 upregulated (BUB1, CCNB1, CDK1, AURKB, KIF11, PBK, NUSAP1) and 1 downregulated (ESR1) hub genes were selected to have significant values as biomarkers. Some of them have been widely reported to be involved in cancer progression. For example, there are many preclinical and clinical studies demonstrating the existence of ESR1 mutations in primary tumors and metastasis lesions, which significantly promoted breast cancer progression [44]; BUB1 was reported to act as an important biomolecule in the regulation of cell cycle in colorectal cancer [45,46].In another word, our credibility of bioinformatic analyses was partially enhanced by the aforementioned publications.

Modulation of gene expression and function by ceRNA regulation attaches great importance to miRNAs and lncRNAs as mentioned above. The web tools miRTarBase and miRNet were then utilized to find 41 potential upstream miRNAs and 113 potential lncRNAs of the biomarkers. The expression level and prognostic value of predicted lncRNAs were further validated by GEPIA database and Kaplan-Meier plotter database.

Moreover, co-expression analysis for all RNA pairs was also performed according to the ceRNA theory. Finally, 18 mRNA-miRNA-lncRNA subnetworks of cervical cancer were acceptable and fresh mRNA-miRNA-lncRNA networks associated with prognosis of cervical cancer were constructed successfully. CCK8, Western blotting and EdU experiments were then performed to validate the RNA pairs in the newly constructed mRNA-miRNA-lncRNA networks. It was proved that ceRNA hypothesis was applied to ESR1/hsa-miR-18a-5p/CCDC144NL-AS1, ESR1/hsa-miR-221-3p/CCDC144NL-AS1, ESR1/hsa-miR-19a-3p/CCDC144NL-AS1 sub-networks.

In order to provide more appropriate prognostic information to help clinicians select patients for accurate therapy, an mRNA-miRNA-lncRNA prognostic signature was established by using the transcriptome data and clinical parameters of cervical cancer. This mRNA-miRNA-lncRNA prognostic signature containing eight mRNA-miRNA-lncRNA subnetworks was further confirmed as an important prognostic indicator in patients with cervical cancer by comparing with the clinical parameters of cervical cancer patients such as pathological stages and age through ROC analysis as well as the univariate and multivariate COX regression analysis.

In conclusion, we successfully investigate some novel ceRNA networks in cervical cancer by way of mRNA-miRNA-lncRNA pattern through successive prediction from mRNAs to lncRNAs. Inspiringly, the identification of mRNA-miRNA-lncRNA prognostic signature may provide some new ideas for clinical prediction of cervical cancer prognosis. Inevitably, despite our successive bioinformatic analyses have attained intriguing findings, there is still a great need for more foundational molecular experiments and large-scale clinical trials to testify the therapeutic values of the potential biomarkers in years to come.

## 5. Conclusion

Our study systematically used public databases to comprehensively analyze mRNA-miRNA-lncRNA expression profiles and prognosis of cervical cancer. A total of 18 mRNA-miRNA-lncRNA subnetworks were identified to be involved in the progression of cervical cancer, as verified by bioinformatic analysis and vitro experiments. And an mRNA-miRNA-lncRNA signature was also established with prognostic value for cervical cancer. It has been displayed by the univariate and multivariate COX regression analysis that the mRNA-miRNA-lncRNA signature happened to be an independent risk indicator for patients in cervical cancer, which may provide some novel ideas for guiding clinicians in making clinical judgments and thereby to improve the outcome of these patients.

## Supplementary Material

Supplemental Material

## Research highlights

- A total of eight biomarkers were found to have prognostic values with cervical cancer.
- A total of 18 mRNA-miRNA-lncRNA subnetworks were found to have functions in the progression of cervical cancer.
- A novel mRNA-miRNA-lncRNA signature was established as an important prognostic indicator in cervical cancer.

## Author statement

Jie Wang: Conceptualization, Methodology, Software, Data curation, Writing-Original draft preparation. Chen Zhang: Visualization, Investigation, Supervision, Software, Writing- Reviewing and Editing.
