# Supplementary material for: Identification and validation of potential mRNA- microRNA- long-noncoding RNA (mRNA-miRNA-lncRNA) prognostic signature for cervical cancer
Source: Bioengineered. 2021 Mar 7;12(1):898–913. doi: 10.1080/21655979.2021.1890377 (PMC8806317; doi:10.1080/21655979.2021.1890377)
Supplement: Supplemental Material [file KBIE_A_1890377_SM9464.docx]

Supplementary Figure 1 (A) The top twenty enriched molecular function (MF) and cellular component (CC) of the upregulated DE-mRNA. (B) The top twenty enriched molecular function (MF) and cellular component (CC) of the downregulated DE-mRNA.


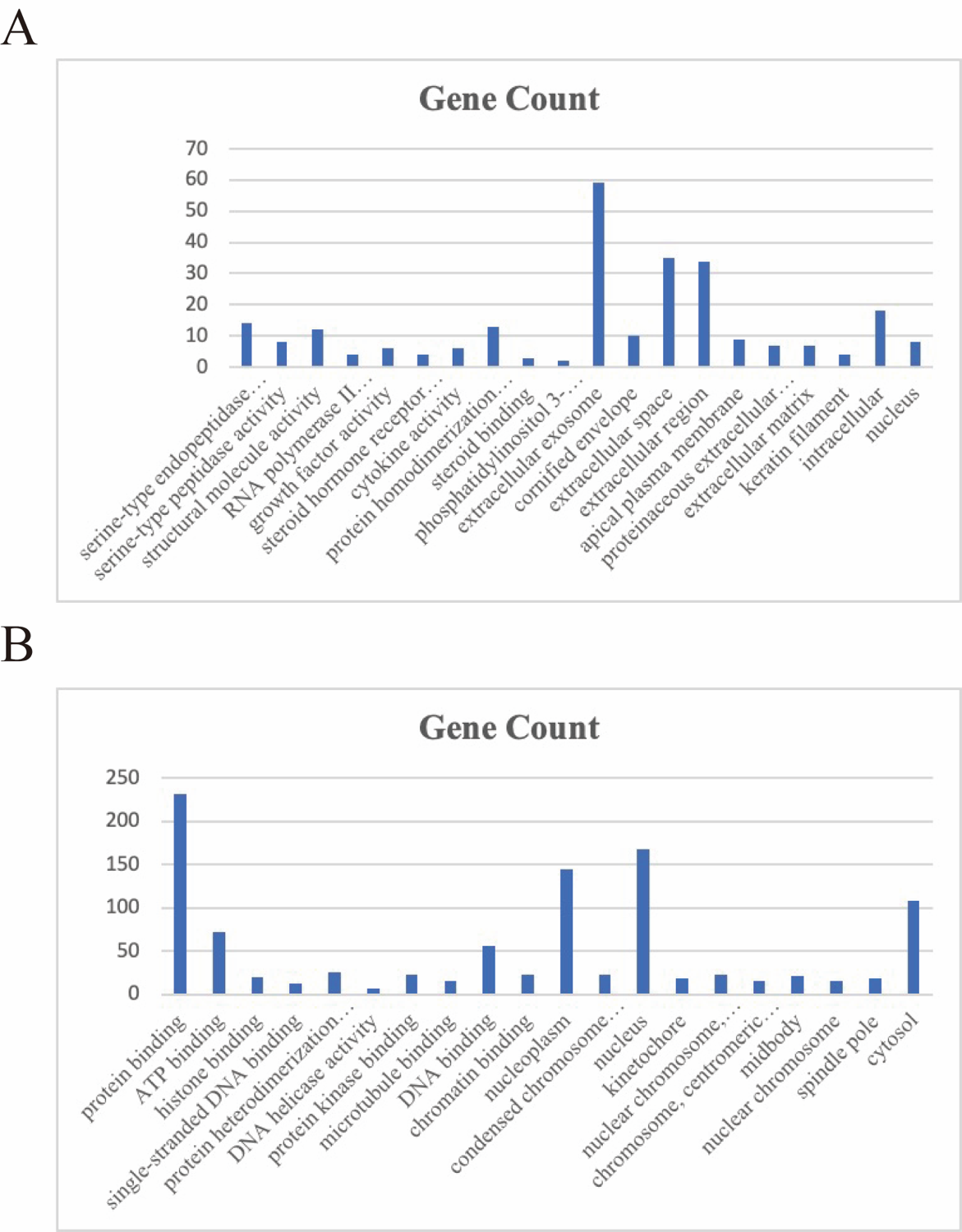


Supplementary Figure 2 (A) The PPI network of the upregulated DE-mRNA. (B) The PPI network of the downregulated DE-mRNA.


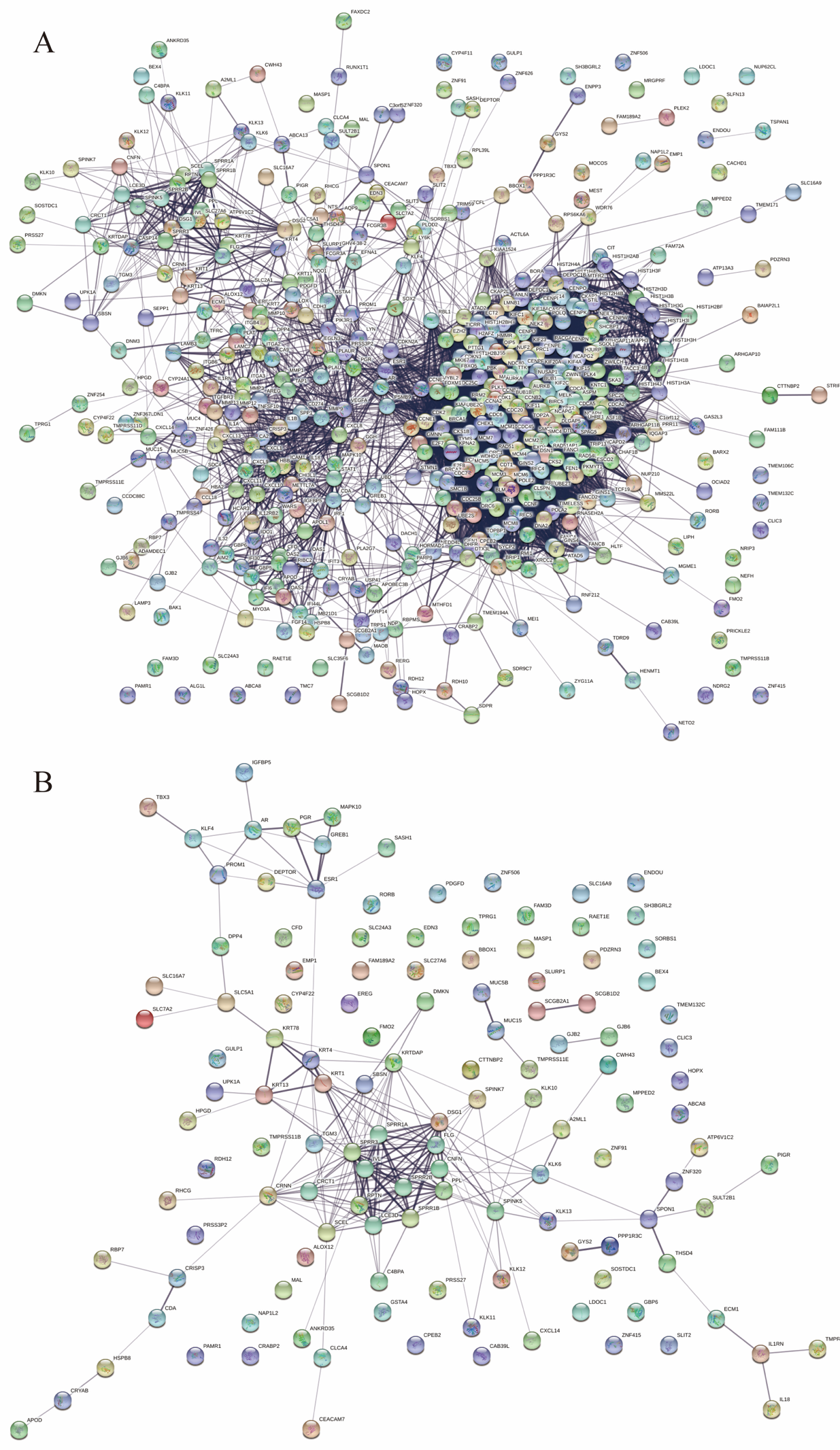
Supplementary Figure 3(A-B) The potential lncRNAs of the key miRNAs


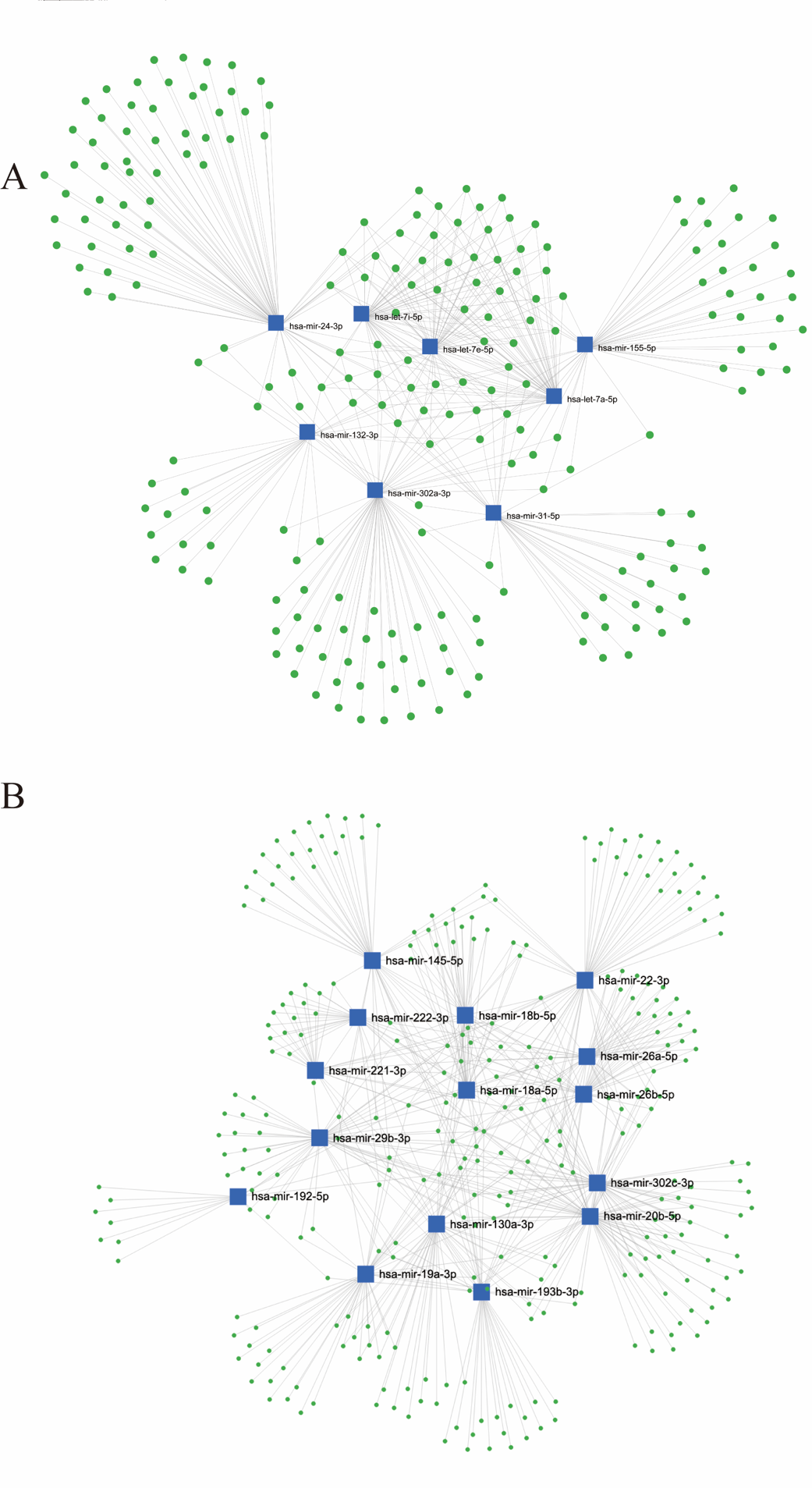


Supplementary table 1. 161 upregulated and 242 downregulated DE-mRNAs

| downregulated genes(161) | upregulated genes(242) |
| --- | --- |
| CRNN  CRISP3  TMPRSS11B  SPINK7  MAL  FAM3D  SPINK5  ENDOU  CLCA4  UPK1A  SPRR3  THSD4  KRT4  EDN3  RDH12  LCE3D  CRCT1  PIGR  ALOX12  KRT1  SBSN  KRTDAP  CLIC3  PPP1R3C  GREB1  TPRG1  SLURP1  CNFN  BBOX1  KLK12  CEACAM7  FMO2  KRT13  A2ML1  RHCG  KLK11  RPTN  HPGD  SOSTDC1  MUC5B  PRSS27  CYP4F22  KLK13  IVL  TMPRSS11E  ESR1  AR  SCEL  DMKN  DSG1  TGM3  CWH43  FLG  CRABP2  CRYAB  TMPRSS11D  SPRR1A  HOPX  CXCL14  GYS2  SLC5A1  GJB6  SULT2B1  APOD  EREG  SPRR1B  EMP1  PGR  GBP6  SCGB2A1  CDA  SLC16A9  GJB2  PROM1  LOC441178  KLK10  KLK6  MPPED2  ATP6V1C2  MUC15  TBX3  SCGB1D2  DPP4  CTTNBP2  IL18  ANKRD35  ZNF506  PRSS3P2  HSPB8  CFD  FAM189A2  TMEM132C  ECM1  SPRR2B  KRT78  ZNF667-AS1  PDGFD  SLC24A3  KLF4  SH3BGRL2  SLC7A2  C4BPA  SPON1  RAET1E  SLC27A6  DEPTOR  BEX4  PAMR1  ZNF91  ZNF415  SLC16A7  PPL  RBP7  LDOC1  GULP1  IGFBP5  RORB  MAPK10  MASP1  GSTA4  SASH1  CPEB2  PDZRN3  ZNF320  SORBS1  NAP1L2  ABCA8  SLIT2  CAB39L  IL1RN  TGFBR3  SEPP1  RUNX1T1  ENPP3  ZNF426  SDR9C7  RBPMS  MRGPRF  TPTEP1  DNM3  NDRG2  RERG  AREG  MAOB  NDP  METTL7A  SDPR  FAXDC2  FGF14  ZNF626  ARHGAP10  CACHD1  BARX2  RPS6KA6  DACH1  PRICKLE2  TRPS1  SLIT3  PIK3R1  ZNF254  LRRK2 | \| DNMT1 \| \| --- \| \| H2AFZ \| \| MTFR2 \| \| CCNE1 \| \| SNORD30 \| \| MB21D1 \| \| NRIP3 \| \| HCP5 \| \| POLA2 \| \| LOX \| \| CXCL13 \| \| RMI1 \| \| HIST1H4L \| \| SLC6A10PB \| \| HIST1H3A \| \| IFIT3 \| \| IL12RB2 \| \| TMEM106C \| \| CDC25B \| \| PKMYT1 \| \| CIT \| \| CCDC88C \| \| RFC5 \| \| BIRC5 \| \| LYN \| \| IL32 \| \| MSL3P1 \| \| LOC100131860 \| \| MTHFD1 \| \| HIST1H4J \| \| SMC2 \| \| ITGB4 \| \| STAT1 \| \| ZNF367 \| \| KIF4B \| \| FANCB \| \| CCNA2 \| \| EFNA1 \| \| PLEK2 \| \| IGK \| \| MMS22L \| \| GINS4 \| \| SLC6A10PB \| \| NEFH \| \| AQP9 \| \| NCAPD2 \| \| OIP5 \| \| HIST1H2BJ \| \| BAIAP2L1 \| \| PLAUR \| \| VEGFA \| \| CCNF \| \| LOC100288637 \| \| FBXO5 \| \| ZWILCH \| \| LY6K \| \| FCGR3A \| \| KRT7 \| \| UBE2S \| \| BORA \| \| MGME1 \| \| NUP62CL \| \| TMEM171 \| \| CD274 \| \| IFI6 \| \| IFI44L \| \| MUC4 \| \| AURKB \| \| CDT1 \| \| NEMP1 \| \| ACTL6A \| \| BAK1 \| \| OAS1 \| \| SOX2 \| \| TDRD9 \| \| CHAF1B \| \| HENMT1 \| \| MAD2L1 \| \| PARP9 \| \| OAS2 \| \| ATP13A3 \| \| RBL1 \| \| HLTF \| \| SDC4 \| \| PSMB9 \| \| PSMB9 \| \| PSMB9 \| \| C1orf112 \| \| GINS2 \| \| SLC35F6 \| \| TACC3 \| \| OCIAD2 \| \| E2F8 \| \| TMC7 \| \| TNFSF10 \| \| ITGA2 \| \| MCM3 \| \| TOPBP1 \| \| CDCA8 \| \| GGH \| \| MCM10 \| \| STRIP2 \| \| CYP4F11 \| \| CA2 \| \| KIAA0101 \| \| FANCD2 \| \| ZYG11A \| \| WARS \| \| TMPRSS4 \| \| IL1A \| \| MCM7 \| \| HIST1H2BF \| \| ITGA3 \| \| ADAMDEC1 \| \| CDCA5 \| \| CDC25C \| \| NEDD4L \| \| ZWINT \| \| HIST1H3H \| \| HMMR \| \| TAP1 \| \| TAP1 \| \| TAP1 \| \| HIST1H3I \| \| NQO1 \| \| LOC102723407 \| \| RNASEH2A \| \| CKAP2L \| \| DTX3L \| \| GAS2L3 \| \| RIBC2 \| \| CKAP2 \| \| TSPAN1 \| \| RAD54L \| \| CDK2 \| \| UBE2S \| \| PRIM1 \| \| CTCFL \| \| DSN1 \| \| HJURP \| \| DHFR \| \| DEPDC1B \| \| HIST1H3H \| \| LOC1720 \| \| CENPU \| \| DHFR \| \| RACGAP1 \| \| TRIP13 \| \| ARHGAP11B \| \| HIST1H2BH \| \| NEIL3 \| \| LAMB3 \| \| FEN1 \| \| CENPW \| \| CA9 \| \| MCM6 \| \| RNF212 \| \| CENPE \| \| GMNN \| \| SLC2A1 \| \| ALG1L \| \| IFI30 \| \| NDC80 \| \| HIST1H1B \| \| PARP14 \| \| CXCL11 \| \| CKS1B \| \| STMN1 \| \| CENPN \| \| HIST1H3G \| \| MMP9 \| \| RDH10 \| \| MYO3A \| \| IL1B \| \| HCAR3 \| \| CYP24A1 \| \| FANCA \| \| HIST2H3D \| \| HIST2H3D \| \| HIST2H4B \| \| HIST2H4B \| \| HIST1H2AB \| \| MEI1 \| \| CKS1B \| \| RAD51 \| \| ICAM1 \| \| PCNA \| \| BRCA1 \| \| GEN1 \| \| IRF1 \| \| OAS3 \| \| FCGR3B \| \| SGO1 \| \| BLM \| \| UHRF1 \| \| KRT17 \| \| KIFC1 \| \| SKA3 \| \| HBA2 \| \| HBA2 \| \| CENPO \| \| TCF19 \| \| ATAD5 \| \| HIST2H3D \| \| C3orf52 \| \| CKS2 \| \| MEST \| \| PLAU \| \| TCF19 \| \| TCF19 \| \| PRR11 \| \| EZH2 \| \| FAM72A \| \| SLFN13 \| \| LIPH \| \| UBE2C \| \| HIST2H4B \| \| HIST2H4A \| \| NUP210 \| \| BRCA2 \| \| PLK4 \| \| CASP14 \| \| TFRC \| \| TRIM59 \| \| SPC25 \| \| USP41 \| \| PLAT \| \| ESCO2 \| \| NCAPH \| \| FAM72A \| \| LAMP3 \| \| NCAPG2 \| \| DNA2 \| \| TK1 \| \| SMC4 \| \| UBD \| \| NETO2 \| \| MOCOS \| \| UBD \| \| TIMELESS \| \| CHEK1 \| \| FAM72A \| \| RPL39L \| \| KPNA2 \| \| XRCC2 \| \| ATAD2 \| \| KPNA2 \| \| CDC20 \| \| MCM8 \| \| DIAPH3 \| \| EGLN3 \| \| CXCL1 \| \| CDC45 \| \| KIF15 \| \| PLA2G7 \| \| POLE2 \| \| CDCA2 \| \| HORMAD1 \| \| LMNB1 \| \| SPAG5 \| \| TICRR \| \| KRT17 \| \| ORC6 \| \| HELLS \| \| HIST1H3F \| \| HIST1H3B \| \| PLOD2 \| \| GBP5 \| \| MCM5 \| \| KNTC1 \| \| CXCL9 \| \| CLSPN \| \| KIFC1 \| \| WDR76 \| \| SHCBP1 \| \| ORC1 \| \| CHI3L1 \| \| KIF18A \| \| PTTG1 \| \| IQGAP3 \| \| PBK \| \| DSG2 \| \| MCM4 \| \| BUB1 \| \| CDC7 \| \| RRM2 \| \| ABCA13 \| \| BUB1B \| \| RAD51AP1 \| \| GINS1 \| \| FAM72A \| \| PLK1 \| \| CCNB1 \| \| MYBL2 \| \| DEPDC1 \| \| CEP55 \| \| KIF20A \| \| TTK \| \| HBB \| \| APOL1 \| \| MKI67 \| \| RFC4 \| \| NCAPG \| \| ITGB6 \| \| CDH3 \| \| NEK2 \| \| WDHD1 \| \| FANCI \| \| CENPK \| \| KIAA1524 \| \| KIF23 \| \| APOBEC3B \| \| POLQ \| \| AURKA \| \| UBE2T \| \| PRC1 \| \| KIF11 \| \| CCNE2 \| \| CXCL8 \| \| CENPI \| \| ASF1B \| \| CLDN1 \| \| MMP13 \| \| TYMS \| \| CDKN3 \| \| ARHGAP11A \| \| BRIP1 \| \| LAMC2 \| \| KIF14 \| \| KNL1 \| \| MCM2 \| \| AIM2 \| \| E2F7 \| \| CCL18 \| \| CCNB2 \| \| MMP10 \| \| NTS \| \| KIF2C \| \| KIF4A \| \| EXO1 \| \| MIR15A \| \| ECT2 \| \| FOXM1 \| \| NUSAP1 \| \| CDK1 \| \| KIAA0101 \| \| MMP3 \| \| DLGAP5 \| \| ANLN \| \| CENPF \| \| CLSPN \| \| ASPM \| \| STIL \| \| MELK \| \| TOP2A \| \| CXCL10 \| \| NUF2 \| \| DTL \| \| CDC6 \| \| IDO1 \| \| TPX2 \| \| FAM111B \| \| SPP1 \| \| CDKN2A \| \| MMP1 \| \| SMC1B \| \| SYCP2 \| \| MMP12 \| |

Supplementary table 2. Top 20 hub genes based on PPI network

| upregulated hub genes | downregulated hub genes |
| --- | --- |
| \| ASPM \| \| --- \| \| CCNB1 \| \| BUB1 \| \| CDK1 \| \| BUB1B \| \| CCNB2 \| \| CCNA2 \| \| NCAPG \| \| RRM2 \| \| AURKB \| \| KIF11 \| \| TTK \| \| PBK \| \| NUSAP1 \| \| TOP2A \| \| MELK \| \| KIF20A \| \| KIF23 \| \| TPX2 \| \| UBE2C \| | \| SPRR3 \| \| --- \| \| LCE3D \| \| IVL \| \| SPRR1A \| \| RPTN \| \| KRTDAP \| \| FLG \| \| CNFN \| \| CRCT1 \| \| PPL \| \| SCEL \| \| CRNN \| \| SBSN \| \| KRT4 \| \| KRT13 \| \| SPINK5 \| \| KRT1 \| \| TGM3 \| \| ESR1 \| \| KRT78 \| |

Supplementary table 3. Expression pattern and survival analysis for top hub genes

| Hub gens | Expression pattern | Prognosis partern |
| --- | --- | --- |
| CCNB1 | Up | Good |
| BUB1 | Up | Good |
| CDK1 | Up | Good |
| AURKB | Up | Good |
| KIF11 | Up | Good |
| PBK | Up | Good |
| NUSAP1 | Up | Good |
| ESR1 | Down | Poor |

Supplementary table 4. A total of 41 potential key miRNAs were predicted to regulate seven biomarkers

| mRNA | miRNA |
| --- | --- |
| CCNB1 | hsa-miR-132-3p |
| CCNB1 | hsa-miR-212-3p |
| CCNB1 | hsa-miR-548b-3p |
| CCNB1 | has-mir-199a-5p |
| CCNB1 | hsa-miR-410-3p |
| BUB1 | hsa-miR-450a-1-3p |
| BUB1 | hsa-miR-10b-3p |
| CDK1 | hsa-miR-31-5p |
| CDK1 | hsa-miR-663a |
| CDK1 | hsa-miR-24-3p |
| CDK1 | hsa-miR-663a |
| CDK1 | hsa-miR-31-5p |
| CDK1 | hsa-miR-31-5p |
| CDK1 | hsa-miR-24-3p |
| CDK1 | hsa-miR-302a-3p |
| AURKB | hsa-miR-24-3p |
| AURKB | mmu-miR-204-5p |
| AURKB | hsa-miR-155-5p |
| AURKB | hsa-let-7a-5p |
| AURKB | hsa-let-7e-5p |
| AURKB | hsa-let-7i-5p |
| KIF11 | / |
| PBK | hsa-miR-216b-5p |
| NUSAP1 | hsa-miR-574-5p |
| ESR1 | hsa-miR-302c-3p |
| ESR1 | hsa-miR-206 |
| ESR1 | hsa-miR-193b-3p |
| ESR1 | hsa-miR-18b-5p |
| ESR1 | hsa-miR-18a-5p |
| ESR1 | hsa-miR-22-3p |
| ESR1 | hsa-miR-19a-3p |
| ESR1 | hsa-miR-29b-3p |
| ESR1 | hsa-miR-20b-5p |
| ESR1 | hsa-miR-221-3p |
| ESR1 | hsa-miR-222-3p |
| ESR1 | hsa-miR-130a-3p |
| ESR1 | hsa-miR-26a-5p |
| ESR1 | hsa-miR-145-5p |
| ESR1 | hsa-miR-26b-3p |
| ESR1 | mmu-miR-125b-1-3p |
| ESR1 | hsa-miR-192-5p |
| ESR1 | hsa-miR-335-3p |

Supplementary table 5. Univariate Cox regression to the expression profiles of the18 RNAs

| Name | p-value | Hazard ratio |
| --- | --- | --- |
| ESR1 | <0.001 | 1.106(1.023-1.165) |
| FTX | 0.007 | 0.834(0.768-0.871) |
| MALAT1 | 0.008 | 0.817(0.754-0.841) |
| CCDC144NL-AS1 | <0.001 | 1.202(1.068-1.287) |
| LINC01089 | <0.001 | 1.187(0.843-0.921) |
| PSMA3-AS1 | <0.001 | 1.185(1.045-1.270) |
| RBPMS-AS1 | <0.001 | 1.152(1.064-1.277) |
| MEG3 | 0.006 | 1.216(1.109-1.286) |
| LINC01278 | 0.005 | 0.851(0.799-0.896) |
| SH3BP5-AS1 | 0.008 | 0.847(0.829-0.915) |
| MIR4697HG | <0.001 | 1.109(1.016-1.203) |
| CRNDE | <0.001 | 1.219(1.073-1.308) |
| CKMT2-AS1 | 0.004 | 0.891(0.876-0.955) |
| LINC00467 | 0.007 | 0.821(0.837-0.967) |
| ZEB1-AS1 | <0.001 | 1.157(1.023-1.277) |
| hsa-miR-18a-5p | <0.001 | 1.208(1.035-1.296) |
| hsa-miR-19a-3p | <0.001 | 1.195(1.021-1.365) |
| hsa-miR-221-3p | <0.001 | 1.201(1.107-1.328) |

Supplementary table 6. Detailed components of PCR reaction solution

| Components | Usage | Final concentrations |
| --- | --- | --- |
| SYBR Premix Ex Taq (2×) | 25ul | 1× |
| PCR Forward Primer(10uM) | 1ul | 0.2uM |
| PCR Reverse Primer(10uM) | 1ul | 0.2uM |
| ROX Reference Dye (50×) | 0.2ul | 0.2× |
| DNA(<100ng) | 4ul |  |
| dH2O | 18.8ul |  |
| Total | 50ul |  |

Supplementary table 7. Detailed procedure of PCR

| Stage | Step | Temperature | Times |
| --- | --- | --- | --- |
| Holding stage | step1 | 95◦C | 30 seconds |
| Cycling stage  (40 cycles) | step1 | 95◦C | 3 seconds |
|  | step2 | 60◦C | 30 seconds |
| Melt curve stage |  | | |
